# Perfectionism, Mood States, and Choking in Asian University Baseball Players under Pressure during a Game

**DOI:** 10.3390/ijerph182312856

**Published:** 2021-12-06

**Authors:** Sang-Jin Yoon, Kazunori Irie, Jun-Ho Lee, Sea-Mi Lim

**Affiliations:** 1 Sport Coaching Science, Graduate School of Health and Sport Science, Nippon Sport Science University, Tokyo 158-8508, Japan; ysangj25@gmail.com (S.-J.Y.); irie@nittai.ac.jp (K.I.); 2 Geumjeong District Council, Busan 46274, Korea; lotte8895@daum.net; 3 Department of Physical Education, Pusan National University, Busan 46241, Korea

**Keywords:** baseball, university baseball players, mood states, choking, perfectionism, mediating effect

## Abstract

The purpose of this study was to investigate relationships among mood states, perfectionism, and choking, and to identify a mediating effect of perfectionism on the relationship between mood states and choking experienced by Asian university baseball players in extremely stressful situations during a game. Data collected from a total of 209 male university baseball players were analyzed using SPSS 21 and AMOS 21 statistical software. The mean age of study subjects was 20.25 years. Results are as follows. First, mood states had a positive influence on perfectionism. Second, mood states had no significant influence on choking. Third, perfectionism had a positive influence on choking. Lastly, perfectionism had a complete mediating effect on the relationship between mood states and choking. The study findings will provide basic data to relieve athletes’ psychological burdens, and prevent manifestations of extreme perfectionism and choking, which can ultimately help athletes maintain high self-control of their mood states and perfectionism for better performance.

## 1. Introduction

Baseball is a highly popular elite and leisure sport in Korea and Japan [1]. Baseball requires high concentration over long periods during play, and players can only perform well when they are physically and mentally healthy; findings indicate correlations between baseball players’ mood states and their performance during a game [2,3].

For Asian university baseball players, every game is an audition for any possible scouts for potential professional players, and exceptional athletic capability during every game is critical in determining amateur players’ future career paths [4]. Advancement to a professional league is the goal of every university baseball player, and the pressure can have varying impacts on their mood states during games [5].

Players frequently experience negative moods, such as tension and confusion, when they set high standards they do not believe they can meet, or when they think others believe they cannot meet them [5,6]. Such negative mood states have detrimental influence on athletes themselves, and their relationships with colleagues, coaches, and parents [6]. Accordingly, it can be said that a multidimensional approach to understanding how moods manifest in performance during competition can provide very important perspectives [7].

In this direction, sports psychologists confirm that mood states influence performance in competitive environments such as sports [8,9,10]. Amateur athletes face constant pressure to achieve professional status, and this extreme focus can trigger potentially disruptive perfectionism; perfectionism, in turn, affects goal achievement, motivations, and motor performance [11]. Therefore, understanding perfectionism in university athletes plays an important role in explaining its adaptive (positive) traits, such as improving athletic skills, boosting motivation, and enhancing performance, as well as its more maladaptive aspects, such as lower self-confidence and poorer performance [12,13].

However, athletes experience choking under pressure in a competitive environment [14]. The choking is defined as ‘showing inferior performance even in the situation where rewards are given for performance at the highest level’ [15] or ‘dramatic performance decline’ occurring in stressful situations [16,17,18,19]. In prior research, athletes who often face performance pressure have vivid feelings on the phenomenon and fear of choking under pressure [5,20], and skill decrements under pressure include not just simple poor performance, but also a form of paralysis that can cause athletes to perform worse than they are actually capable of [15,20]. Hall [21] found that athletes have quite individual and subjective perceptions regarding the phenomenon of choking under pressure. However, there is still a lack of sophisticated understanding about the performance failure phenomenon of choking under pressure under critical situations [22,23]

Researchers propose two representative mechanisms to explain choking, distraction theory and explicit monitoring (or self-focus) theory [14,24]. In distraction theory, the pressure during execution can increase self-awareness, which increases attention to executing a skilled performance. On the contrary, the crux of explicit monitoring theory is how performers can control their own mood states and attention in high-pressure situations [14]. Opinions differ on which of these two theories better explains the choking phenomenon, and studies are needed on choking in different tasks contexts, as well as according to skill level, individual mood, and sensitivities to perfectionism. Results from such research should provide useful knowledge to help athletes and coaches train to overcome the choking that can occur in pressure situations.

Toward the aim of offering clearer data on the phenomenon of choking among university athletes, the purpose of this study was to empirically investigate relationships among mood states, perfectionism, and choking, and to identify any mediating effect of perfectionism on the relationship between mood states and choking in Asian university baseball players in high-pressure game situations. We investigated these relationships by testing the following hypotheses.

**Hypothesis** **1** **(H1).** *Mood states in extremely stressful situations affect perfectionism (p < 0.05)*.

**Hypothesis** **2** **(H2).** *Mood states in extremely stressful situations affect choking (p < 0.05)*.

**Hypothesis** **3** **(H3).** *Perfectionism in extremely stressful situations affects choking (p < 0.05)*.

**Hypothesis** **4** **(H4).** *Perfectionism in extremely stressful situations has a mediating effect on the relationship between mood states and choking (p < 0.05)*.

## 2. Materials and Methods

### 2.1. Participants

Convenience sampling method was used to select 223 baseball players that were enrolled at universities in Korea and Japan in the period between November 2019 and February 2020 for data collection. We administered to the students a survey that had been originally written in Korean, and translated into Japanese; a specialized translation company certified the accuracy of the Japanese translation against the original document. The Ethics Committee of Nippon Sports Science University approved this study (019-H132).

Before signing the written consent, all selected university baseball players were briefed on the scope and objectives of the study, then they completed the questionnaires during their free time. Between ten and twenty participants were engaged in each survey session, in a process that cumulatively collected data from a total of 223 baseball players. The average time to complete the questionnaire per session was approximately 30 min, with 10 min for the students to give their own oral definitions of the phenomenon of choking, and 20 min to complete the questionnaires. We offered during each session to answer any questions the athletes had about survey content, and we reconfirmed that their participation in the study was voluntary. After we excluded 14 participants whose surveys indicated that they had never experienced choking during a pressure situation, 209 survey responses remained for analysis. Table 1 presents the general characteristics of these 209 subjects.

### 2.2. Data Processing

For the questionnaire in this study, survey respondents rated each item on a 5-point Likert scale ranging from 1 (*I strongly disagree*) to 5 (*I strongly agree*); except the demographic characteristics, Table 2 presents the survey items. We analyzed the collected data using SPSS 21 (IBM, Armonk, NY, USA) and AMOS 21 (IBM, Chicago, IL, USA) in accordance with the following procedure to ensure survey validity and reliability. First, we conducted frequency analysis of the students’ general demographic characteristics (i.e., background variables). Second, survey content validity had been evaluated before the study commenced by a consultation committee of three experts with PhDs in physical education. Third, we conducted confirmatory factor analysis (CFA) to calculate the construct validity of the survey, and calculated reliability though internal consistency estimation (Cronbach’s α coefficient) for a derived subfactors. We tested convergent validity using average variance extracted (AVE) and construct reliability (CR) coefficients, where convergent validity is established if AVE is 0.5 or higher, and CR is 0.7 or higher [25].

Fourth, we conducted Pearson’s correlation analysis to analyze the relationships between subfactors before testing the hypotheses (*p* < 0.01); the relationship between two subfactors is considered strong when r is 0.7 or higher. Fifth, we ran structural equation modeling (SEM) to test the study hypotheses. According to previous researchers, SEM requires establishing clear interpretation criteria for research model goodness-of-fit considering model parsimony and sensitivity to sample size [25,26,27]. To this end, Kline [28], Hooper, Coughlan, and Mullen [29], No [25], and Kim et al. [27] established the following criteria as indicating good model fit: SRMR (standardized root mean square residual) = 0.08 or less, IFI (incremental fit index), TLI (Tucker–Lewis index), CFI (comparative fit index) = 0.9 or higher, and RMSEA (root mean square error of approximation) = 0.1 or less. Lastly, we tested goodness-of-fit of the complete mediating model, and performed bootstrapping analysis to investigate the mediating effect of perfectionism on the relationship between mood states and choking.

### 2.3. Extremely Stressful Situations

To help subjects fully understand how we intended “extremely stressful situations”, we spent approximately 10 min discussing the concept with the student athletes in each session before they filled out their questionnaires. “Extremely stressful situations” refers to sudden and abnormal changes in mood states during a game that cause athletes to either underperform or concentrate more intensely. For instance, the bottom of the ninth inning with bases loaded and a score of 3–2 is a critical moment when the losing team’s pitchers, batters, and fielders should perform to the best of their ability. For this study, we identified three or four potential extremely stressful situations that could occur during a game, and described them in detail to the participants. In addition to the oral instruction, the front page of the questionnaire also bore a detailed description of extremely stressful situations, in order to ensure all subjects were fully aware of the definition before participating in the survey.

## 3. Results

### 3.1. Descriptive Statistics

Overall, 209 baseball players from Asian universities (148 Japanese and 61 Koreans) were enrolled in the study. The mean age was 20.25 years, mean career duration was 12.1 years, whereas the pitchers’ mean career duration was 5.6 years.

### 3.2. Mood States

**Figure 1:**
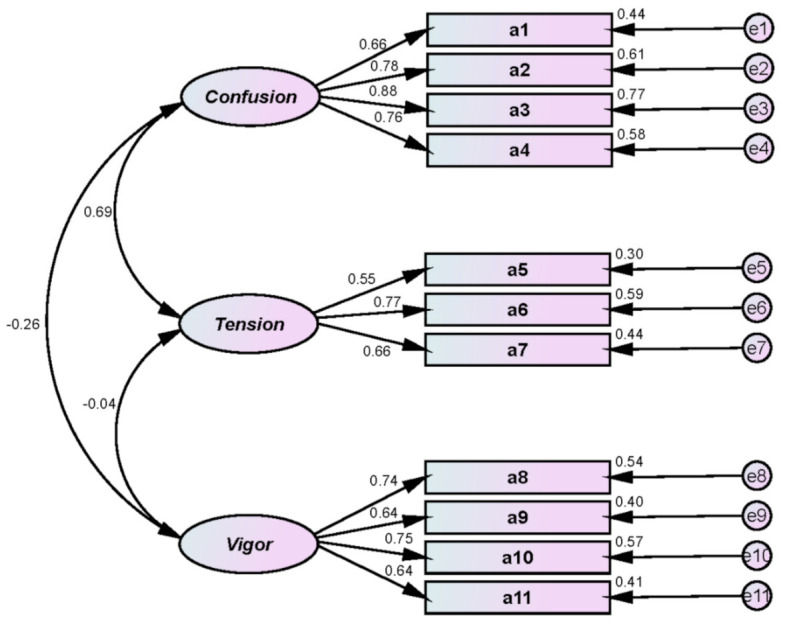
Mood states CFA.

We measured the athletes’ mood states using Park’s [30] modified version of the Profile of Mood States (POMS), which had been revised from the original by McNair, Lorr, and Droppleman [31]. As Table 2 shows, mood states were measured with a total of 11 questions in 3 subfactors: 4 for confusion; 3 for tension; and 4 for vigor. Table 3 and Figure 1 display the CFA results.

First, all indexes indicated adequate goodness-of-fit: χ^2^(df) = 95.788(41)/*p* < 0.001; SRMR = 0.066; IFI = 0.935; TLI = 0.912; CFI = 0.934; RMSEA = 0.080. Second, survey reliability was confirmed: Cronbach’s α = 0.877 for confusion; 0.894 for tension; and 0.874 for vigor. Third, CR and AVE findings confirmed the convergent validity of the survey tool: CR = 0.938 for confusion; 0.829 for tension; and 0.829 for vigor; and AVE = 0.578 for confusion; 0.618 for tension; and 0.550 for vigor.

### 3.3. Perfectionism

**Figure 2:**
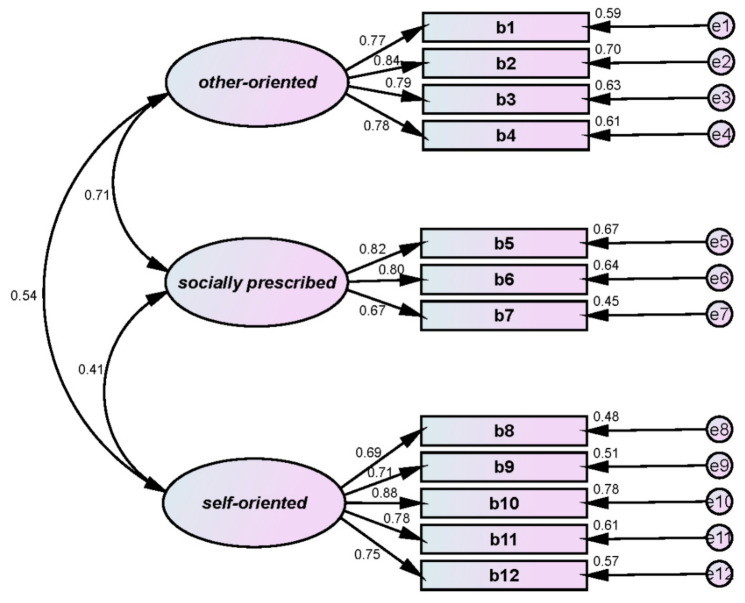
Perfectionism CFA.

We measured perfectionism using Kim’s [12] modified version of the Multi-dimensional Perfectionism Scale (MPS), originally developed by Hewitt and Flett [32] to investigate perfectionism in university rugby players. Table 2 shows that the perfectionism scale comprised 12 questions in 3 subfactors: 4 for other-oriented; 3 for socially prescribed; and 5 for self-oriented. Table 3 and Figure 2 display the CFA results.

First, all indexes met the goodness-of-fit requirements: χ^2^(df) = 126.008(50)/*p* < 0.001; SRMR = 0.060; IFI = 0.944; TLI = 0.926; CFI = 0.944; RMSEA = 0.085. Second, reliability of the survey tool was confirmed: Cronbach’s α = 0.872 for other-oriented; 0.812 for socially prescribed; and 0.873 for self-oriented. Third, CR and AVE results confirmed convergent validity: CR = 0.833 for other-oriented; 0.792 for socially prescribed; and 0.851 for self-oriented; and AVE = 0.555 for other-oriented; 0.559 for socially prescribed; and 0.535 for self-oriented.

### 3.4. Choking

We investigated choking among the student athletes using Park’s [5] modification of Murayama and Sekiya’s [33] original 77-question choking scale. Table 2 shows that choking was measured with 10 questions on 3 subfactors: 3 for anxiety-related accidents; 3 for self-focus and motor control; and 4 for cognitive, emotional, and perceptual confusion.

**Figure 3:**
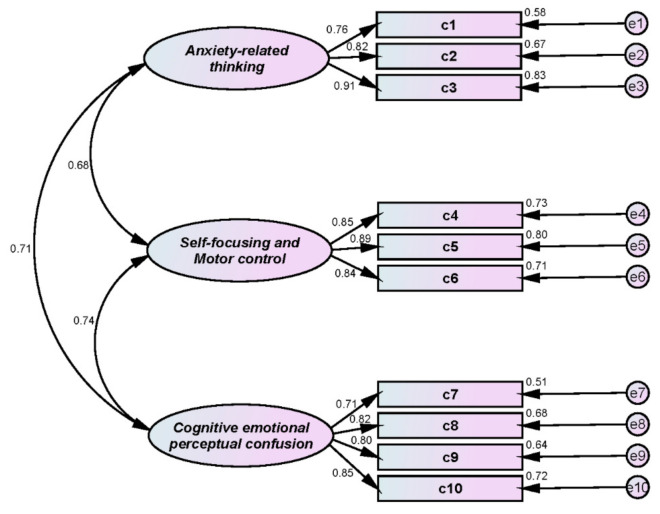
Choking CFA.

First, we conducted CFA to verify the construct validity of the scale, and results are shown in Table 3 and Figure 3; all indexes met goodness-of-fit requirements: χ^2^(df) = 93.005(32)/*p* < 0.001; SRMR = 0.047; IFI = 0.957; TLI = 0.939; CFI = 0.957; RMSEA = 0.096. Second, reliability was confirmed: Cronbach’s α = 0.870 for anxiety-related thinking; 0.896 for self-focusing and motor control; and 0.874 for cognitive, emotional, and perceptual confusion. Third, CR and AVE results confirmed convergent validity: CR = 0.865 for anxiety-related thinking; 0.879 for self-focusing and motor control; and 0.854 for cognitive, emotional, and perceptual confusion; and AVE = 0.682 for anxiety-related thinking; 0.707 for self-focusing and motor control; and 0.595 for cognitive, emotional, and perceptual confusion.

### 3.5. Pearson’s Correlation Analysis

Table 4 presents the Pearson’s correlations for the study constructs. First, confusion, a mood state subfactor, showed statistically significant positive correlations with other-oriented and socially prescribed subfactors of perfectionism (r = 0.406 and 0.387, respectively; *p* < 0.01). Tension, another mood state subfactor, also showed a significant positive correlation with the other-oriented perfectionism subfactor (r = 0.216; *p* < 0.01). Second, tension also had significant positive correlations with all subfactors of choking (r = 0.272, 0.191, and 0.230; all *p* < 0.01). Lastly, the other-oriented perfectionism subfactor showed statistically significant positive correlations with all choking subfactors (r = 0.304, 0.202, and 0.296; all *p* < 0.01). The self-oriented perfectionism subfactor also had significant positive correlations with the choking subfactors of anxiety-related thinking and cognitive, emotional, and perceptual confusion (r = 0.183 and 0.259, respectively; *p* < 0.01). In a preceding study, Kline [28] determined that a correlation coefficient between subfactors of 0.85 or less indicates that there is no multicollinearity between subfactors. Based on this criterion, there was no multicollinearity between subfactors: r = −0.216 to 0.638. Table 5 presents the overall goodness-of-fit findings for the study model, and, below, we discuss the findings for the hypothesis testing.

### 3.6. Hypothesis Testing

With this study, we aimed to investigate relationships among mood states, perfectionism, and choking among a group of male university baseball students from Korea and Japan in moments of what we called extremely stressful situations during games. We also were aiming to identify a mediating effect of perfectionism on the relationship between mood states and choking. We tested the hypotheses using SEM analysis, and Table 5 shows these results.

First, analysis of the relationship between mood states and perfectionism showed that mood states had a statistically significant positive influence on perfectionism with a path coefficient of 0.111 (t = 2.059, *p* < 0.05). Therefore, H1 was accepted. Second, analysis of the relationship between mood states and choking revealed no significant influence of mood states on choking, with a path coefficient of −0.031 (t = −0.971). Therefore, H2 was rejected. Third, analysis of the relationship between perfectionism and choking revealed a significant positive influence of perfectionism on choking, with a path coefficient of 0.538 (t = 3.808, *p* < 0.01). Therefore, H3 was accepted.

We also analyzed goodness-of-fit of the complete mediation model, and conducted bootstrapping to analyze the mediating effect of perfectionism on the relationship between mood states and choking. Table 6 shows that the complete mediation model met the goodness-of-fit requirements: χ^2^(df) = 57.290(24)/*p* = 0.001; SRMR = 0.066; IFI = 0.940; TLI = 0.908; CFI = 0.938; RMSEA = 0.082. Bae [26] established that if the difference between an incomplete and a complete mediation model is χ^2^= 3.84 or less at α = 0.05, and the degree of freedom is 1 or less, mediation is complete. Because the difference between the two models here was estimated at Δχ^2^ = 1.095 and Δdf = 1, complete mediation (indirect effect) was confirmed. To verify the significance of an indirect effect, we conducted bootstrapping with a repetition frequency of 2000 times, and a bias-corrected confidence interval of 95%, and the results showed a statistically significant (complete) mediation effect at *p* = 0.001. That is, perfectionism had a complete mediating effect on the relationship between mood states and choking, and H4 was accepted.

## 4. Discussion

The purpose of the present study was to investigate relationships among mood states, perfectionism, and choking perceived by Asian university baseball players in extremely stressful situations during a game, and identify the mediating effect of perfectionism on the relationship between mood states and choking. Below, we present a discussion of our findings.

First, mood states had a positive effect on perfectionism. Sports inevitably involve competition, which triggers a wide range of mood states in athletes, and researchers have studied mood states to predict athletes’ behaviors, tendencies, and performance [34]. In addition, mood is an important factor to improve athletes’ motor performance ability, as well as their athletic performance [35,36]. We confirmed the influence of mood states on perfectionism in the present study consistent with preceding studies regarding the relationship between mood states and behavior tendencies.

For instance, researchers have identified perfectionism as a behavior tendency with both positive and negative impacts [37,38,39,40,41,42], and here, we determine that mood states can determine a perfectionist personality. The double-edged sword of perfectionism is associated with motor performance ability with documented positive and negative impacts [40,43]. When this personality tendency trends toward the positive, perfectionism shows positive, rather than negative, impacts on motor performance ability. Follow-up researchers could investigate the impacts of predisposing factors other than mood states that can control perfectionism, and contribute to the development of psychological coaching methods to improve performance.

Second, mood states had no significant influence on choking in this study, which could be attributable to individual differences in personality traits [44]. High-pressure situations can lead some players to choke, and can stimulate clutch performance for others, leading to inconsistent findings [45,46,47]. Separately, Gill [48], Gould and Udry [49], Hanin [50,51], Kerr [52], Lazarus [53], and Males and Kerr [54] established that stress, confusion, and tension alone are not sufficient to explain the complicated relationship between mood states and athletes’ motor performance ability. Overall, there are few empirical confirmations of a relationship between mood states and choking, and it could be fruitful to study how individual personality traits affect mood state responses, such as tension and confusion.

Third, perfectionism had a positive influence on choking. Perfectionism is a personality trait characterized by setting excessively high standards for performance, and striving for superior performance to that of others to win a game in competition with others [55]. People with perfectionist tendencies have multidimensional personality traits characterized by overly critical evaluations of their own actions, and excessive sensitivity to mistakes [56,57,58], and perfectionism is visibly evident among athletes. Empirical findings from both domestic and overseas studies on perfectionism have showed that excessively high perfectionist tendencies can cause or aggravate psychopathology-related factors, including depression, tension [59,60,61], stress, fear, and anger [62,63,64,65]. Excessive perfectionism is also closely related to competitive state anxiety, burnout, and exercise stress [11,66,67].

University baseball players strive for perfection during games because these serve as the only window for them to appeal to professional teams, and we believe that obsession with perfection can lead to choking. It is also the case that in Korean society, baseball players are rated on the binary criterion of being either a success or a failure, and this pressure could lead to negative perfectionism. Baseball players who have spent many years pursuing their dream of becoming professional players are likely to have limited options to earn a living if they fail to advance to a professional team, and they can feel guilty about their families’ sacrifices for their dream. These complicated mood states can cause athletes to redouble their efforts, and drive them toward an extreme level of perfectionism. Therefore, for players to perform without the pressure of perfectionism, family members and other people around young athletes lower their expectations for players’ success.

Lastly, perfectionism had a mediating effect on the relationship between mood states and choking. It is well-known that negative mood states, such as tension or confusion, do not necessarily lead to choking, but some athletes experience more serious choking in performance, informally known as the yips. Perfectionism directly affected choking in this study, and had an indirect mediating effect on the impact of mood states on choking.

Previous researchers have established that perfectionism affects athletic performance, and that athletes feel more pressure in real games than they do in practice, triggering heightened emotions [40,68,69]. These results seem meaningful in that mood state and performance are not separable in some athletes: consistent, for instance, with So’s [35] finding that emotional intelligence had a major influence on athletic performance.

The above findings suggest that counseling could protect athletes from manifesting extreme perfectionism, and, in turn, help make choking less likely under pressure, and that increasing the understanding and management of perfectionism, considering the distinct characteristics of baseball players, would be a valuable area for future research. In addition to the control and management of athletes’ psychological conditions, reflection is warranted on whether coaches’ teaching procedures and methods are appropriate, and whether the people around certain athletes, such as family members and relatives, have been careful with them in consideration of the players’ perfectionism. Such reflective attitudes in the people around athletes can help relieve athletes’ psychological burdens, and prevent manifestations of extreme perfectionism and choking, which can ultimately help athletes maintain high self-control of their mood states and perfectionism for better performance.

## 5. Conclusions and Suggestions

The purpose of the present study was to investigate relationships among mood states, perfectionism, and choking, and determine the mediating effect of perfectionism on the relationship between mood states and choking of Asian university baseball players in extremely stressful situations during a game. Based on our research results, we have the following conclusions.

First, mood states had a positive influence on perfectionism. Second, mood states had no significant influence on choking. Third, perfectionism had a positive influence on choking. Lastly, perfectionism had a (complete) mediating effect on the relationship between mood states and choking. In the present study, we reach the conclusion that perfectionism is one of causes that leads to choking in extremely stressful situations. Mood states are simple moods felt under pressure, whereas perfectionism is an athlete’s subjective perception of moods. Under usual situations, mood states do not affect choking or an athlete’s performance. However, if perfectionism is involved in mood states in extremely stressful situations, perfectionism can affect choking directly, and acts as a mediator to allow mood states to affect choking indirectly. This result has never been reported by preceding studies. This evidence strongly suggests that with perfectionism controlled better, choking can be controlled better in competitive situations. Accordingly, perfectionism evaluation can be an important psychological scale of the choking-susceptible athlete to overcome choking.

Future research should be directed towards the identification of other psychological mediator variables that may evoke choking or performance decrements. Extending and classifying our knowledge of potential mediator variables, such as competitive anxiety or state anxiety, which increase the likelihood of choking, can allow us to improve interventions for performance decrement under pressure.

We think that follow-up observation and qualitative research designed to confirm the cause of the derived result will be significantly meaningful for helping athletes overcome choking (yips), or in preventing injury resulting from a sudden performance change.

Comparative studies that include various cultural and situational factors related to Asian university players should also be conducted. Additionally, follow-up studies targeting other nationalities, age-groups, athletic performance levels, and sexes should be conducted.

## Figures and Tables

**Table 1:** Characteristics of participants (n = 209).

Variable	Division	n	%
Gender	Male	209	100
Nationality	Korean	61	29
	Japanese	148	71
Grade	Freshman	68	32.5
	Sophomore	58	27.8
	Junior	45	21.5
	Senior	38	18.2
	Average age (years)	209	20.25
Position	Pitcher	139	66.5
	Fielder	70	33.5
Period	Baseball career(years)	209	12.1
	Pitcher career(years)	139	5.6

**Table 2:** Contents of the questionnaire.

Variables	Index	Question	Total
Background Variables	General characteristics	Gender (1)	6
		Nationality (1)	
		Grade (1)	
		Position (1)	
		Period (2)	
Independent Variables	Mood states	Confusion (4)	11
		Tension (3)	
		Vigor (4)	
Mediating Variables	Perfectionism	Other-oriented (5)	12
		Socially prescribed (4)	
		Self-oriented (3)	
Dependent Variables	Choking	Anxiety-related thinking (3)	10
		Self-focusing and motor control (3)	
		Cognitive, emotional, and perceptual confusion (4)	
Total			39

**Table 3:** Validity and reliability analyses.

Variables		Item	λ	S.E.	C.R.(t)	*p*	SC	AVE	C.R.	ɑ
Mood states	confusion	a1. distracted	1.000				0.660	0.578	0.938	0.877
		a2. woozy	1.122	0.118	9.537	0.001	0.780			
		a3. perplexed	1.435	0.139	10.302	0.001	0.880			
		a4. uncertain	1.134	0.122	9.330	0.001	0.758			
	tension	a5. nervous	1.000				0.548	0.618	0.829	0.894
		a6. agitated	1.445	0.219	6.607	0.001	0.767			
		a7. restless	1.344	0.212	6.330	0.001	0.664			
	vigor	a8. energetic	1.000				0.736	0.550	0.829	0.874
		a9. active	0.978	0.125	7.821	0.001	0.636			
		a10. lively	1.047	0.120	8.755	0.001	0.753			
		a11. cheerful	0.975	0.124	7.838	0.001	0.638			
χ^2^ = 95.788, df = 41, *p* = 0.001, SRMR = 0.066, IFI = 0.935, TLI = 0.912, CFI = 0.934, RMSEA = 0.080										
Perfectionism	other-oriented	b1. People around me expect more than what I am capable of	1.000				0.765	0.555	0.833	0.872
		b2. People around me expect me to be perfect	1.203	0.098	12.237	0.001	0.839			
		b3. My family expect me to be perfect	1.244	0.108	11.563	0.001	0.795			
		b4. People around me expect too much from me	1.104	0.097	11.418	0.001	0.783			
	socially prescribed	b5. People around me will like me when I excel in sports and everything	1.000				0.819	0.559	0.792	0.812
		b6. People around me would think of me as a nice person only if I am successful	1.020	0.092	11.086	0.001	0.802			
		b7. People around me would think of me as competent only if I don’t make a mistake	0.817	0.086	9.450	0.001	0.670			
	self-oriented	b8. I try to be as perfect as possible	1.000				0.691	0.535	0.851	0.873
		b9. It is important for me to be perfect in everything	1.004	0.107	9.359	0.001	0.711			
		b10. I want myself to be perfect	1.447	0.129	11.229	0.001	0.884			
		b11. I have a strong desire to become perfect	1.393	0.136	10.216	0.001	0.783			
		b12. My goal is to be perfect in everything	1.404	0.142	9.853	0.001	0.752			
χ^2^ = 126.008, df = 50, *p* = 0.001, SRMR = 0.060, IFI = 0.944, TLI = 0.926, CFI = 0.944, RMSEA = 0.085										
Choking	anxiety-related thinking	c1. I was concerned about how other people think of me	1.000				0.765	0.682	0.865	0.870
		c2. I couldn’t shake off a mistake and kept thinking of it	1.062	0.087	12.155	0.001	0.818			
		c3. I was worried about and afraid of disappointing other people	1.265	0.096	13.235	0.001	0.909			
	self-focusing and motor control	c4. My decision-making ability was worse than normal due to high pressure	1.000				0.853	0.707	0.879	0.896
		c5. I moved impatiently	1.034	0.064	16.122	0.001	0.893			
		c6. My movement was stiff and not soft	0.983	0.066	14.902	0.001	0.842			
	cognitive, emotional, and perceptual confusion	c7. I felt as if all people watched only me	1.000				0.713	0.595	0.854	0.874
		c8. I became more conscious of the surrounding environment than usual	1.067	0.096	11.139	0.001	0.824			
		c9. I thought that things around me and the environment were against me	1.144	0.106	10.798	0.001	0.797			
		c10. I was engulfed by the atmosphere	1.096	0.096	11.461	0.001	0.851			
χ^2^ = 93.005, df = 32, *p* = 0.001, SRMR = 0.047, IFI = 0.957, TLI = 0.939, CFI = 0.957, RMSEA = 0.096										

**Table 4:** Pearson’s correlations among mood states, perfectionism, and choking.

Variables	Subfactors	1	2	3	4	5	6	7	8	9
Mood states	confusion (1)	1								
	tension (2)	0.519 **	1							
	vigor (3)	−0.216 **	−0.012	1						
Perfectionism	other-oriented (4)	0.406 **	0.216 **	0.002	1					
	socially prescribed (5)	0.387 **	0.096	−0.016	0.597 **	1				
	self-oriented (6)	0.054	0.072	0.008	0.464 **	0.345 **	1			
Choking	anxiety-related thinking (7)	0.101	0.272 **	−0.019	0.304 **	0.021	0.183 **	1		
	self-focusing and motor control (8)	0.087	0.191 **	0.032	0.202 **	0.088	0.131	0.600 **	1	
	cognitive, emotional, and perceptual confusion (9)	0.117	0.230 **	0.047	0.296 **	0.098	0.259 **	0.620 **	0.638 **	1

** *p* < 0.01.

**Table 5:** Path analysis and fit index of the research model.

H	Path			Estimate	S.E.	C.R(t)	Sig.	Result
H1	Mood states	→	Perfectionism	0.111	0.054	20.059	0.039	Accept
H2	Mood states	→	Choking	−0.031	0.032	−0.971	0.332	Reject
H3	Perfectionism	→	Choking	0.538	0.141	30.808	0.001	Accept
Fit Index			χ^2^(df) = 56.195(23)/p = 0.001, SRMR = 0.071, IFI = 0.940, TLI = 0.904, CFI = 0.939, RMSEA = 0.083					

**Table 6:** Mediating effect analysis through bootstrapping.

Path					Bootstrap Estimates 95% Confidence Interval					
					Indirect Effect		Lower		Upper	
Mood states	→	Perfectionism	→	Choking	0.001		0.040		0.234	
Model		χ^2^		df	*p*	SRMR	IFI	TLI	CFI	RMSEA
Complete mediation		57.290		24	0.001	0.066	0.940	0.908	0.938	0.082

Δχ^2^ = 1.095, Δdf = 1.

## Data Availability

The data presented in this study are available from the corresponding author upon reasonable request.
